# Sister chromatid cohesion defects are associated with chromosome instability in Hodgkin lymphoma cells

**DOI:** 10.1186/1471-2407-13-391

**Published:** 2013-08-20

**Authors:** Babu V Sajesh, Zelda Lichtensztejn, Kirk J McManus

**Affiliations:** 1 Manitoba Institute of Cell Biology and the Department of Biochemistry & Medical Genetics, University of Manitoba, ON6010 – 675 McDermot Avenue Winnipeg, Manitoba MB R3E 0V9, Canada

**Keywords:** Hodgkin lymphoma, Chromosome instability, Sister chromatid cohesion, HDLM-2, L-540, RAD21

## Abstract

**Background:**

Chromosome instability manifests as an abnormal chromosome complement and is a pathogenic event in cancer. Although a correlation between abnormal chromosome numbers and cancer exist, the underlying mechanisms that cause chromosome instability are poorly understood. Recent data suggests that aberrant sister chromatid cohesion causes chromosome instability and thus contributes to the development of cancer. Cohesion normally functions by tethering nascently synthesized chromatids together to prevent premature segregation and thus chromosome instability. Although the prevalence of aberrant cohesion has been reported for some solid tumors, its prevalence within liquid tumors is unknown. Consequently, the current study was undertaken to evaluate aberrant cohesion within Hodgkin lymphoma, a lymphoid malignancy that frequently exhibits chromosome instability.

**Methods:**

Using established cytogenetic techniques, the prevalence of chromosome instability and aberrant cohesion was examined within mitotic spreads generated from five commonly employed Hodgkin lymphoma cell lines (L-1236, KM-H2, L-428, L-540 and HDLM-2) and a lymphocyte control. Indirect immunofluorescence and Western blot analyses were performed to evaluate the localization and expression of six critical proteins involved in the regulation of sister chromatid cohesion.

**Results:**

We first confirmed that all five Hodgkin lymphoma cell lines exhibited chromosome instability relative to the lymphocyte control. We then determined that each Hodgkin lymphoma cell line exhibited cohesion defects that were subsequently classified into mild, moderate or severe categories. Surprisingly, ~50% of the mitotic spreads generated from L-540 and HDLM-2 harbored cohesion defects. To gain mechanistic insight into the underlying cause of the aberrant cohesion we examined the localization and expression of six critical proteins involved in cohesion. Although all proteins produced the expected nuclear localization pattern, striking differences in RAD21 expression was observed: RAD21 expression was lowest in L-540 and highest within HDLM-2.

**Conclusion:**

We conclude that aberrant cohesion is a common feature of all five Hodgkin lymphoma cell lines evaluated. We further conclude that aberrant RAD21 expression is a strong candidate to underlie aberrant cohesion, chromosome instability and contribute to the development of the disease. Our findings support a growing body of evidence suggesting that cohesion defects and aberrant RAD21 expression are pathogenic events that contribute to tumor development.

## Background

For over a century, it has been suggested that abnormal chromosome numbers are a pathogenic event that are associated with and drive cancer development [1]. Extensive studies have confirmed that virtually all tumor types, including solid (e.g. colorectal cancer [2]) and liquid (e.g. Hodgkin lymphoma [3]), exhibit genomic instability that often manifests as chromosome instability (CIN). CIN is a dynamic process that is defined by an increased rate at which whole chromosomes or large parts thereof are gained or lost [4], consequently aneuploidy is now employed as a metric for CIN. Conceptually, CIN promotes tumor heterogeneity by increasing numerical or structural chromosomal changes that in turn impacts oncogene and/or tumor suppressor gene copy numbers [5]. Thus, CIN is a driver of the tumorigenic process [6] and often arises through defects in DNA repair, DNA replication or chromosome segregation [2,7-9]. Tumors displaying CIN (i.e. an aneuploid karyotype) are associated with poor prognosis, and the rapid acquisition of multidrug resistance [10-12]. Despite the strong correlation between CIN and tumors, very little is known about the underlying aberrant genes and/or mechanisms that account for the CIN phenotype.

A recent body of evidence has begun to emerge suggesting that aberrant sister chromatid cohesion may be a pathogenic event that underlies CIN and drives tumor development. Sister chromatid cohesion is an evolutionarily conserved biological process that ensures the faithful segregation of genomic DNA from mother to daughter cells. Classically, cohesion serves to prevent premature chromosome segregation during mitosis by tethering newly synthesized sister chromatids together prior to entry into anaphase [13], however it also functions in DNA repair [14] and gene expression [15]. Mitotic cohesion is effected by a quaternary complex referred to as cohesin [13], which is comprised of SMC1A, SMC3, RAD21 and STAG1/STAG2/STAG3. Cohesin is initially loaded onto DNA in G1 by cohesin loaders (NIPBL and MAU2), but isn’t established until S-phase when SMC3 is acetylated by ESCO1 and ESCO2 [16]. Although the exact mechanism (i.e. structure) by which cohesin tethers the sister chromatids is unclear, it is believed to form a ‘ring-like’ structure that encircles the two DNA strands [17]. During the initial stages of mitosis (prophase to metaphase), cohesion is first lost along the length of the chromosome arms but is maintained/protected at the centromeres through a Shugoshin-dependent mechanism [18]. A critical requirement of the metaphase to anaphase transition is the regulated cleavage of the remaining centromeric cohesion prior to chromosome segregation. Cohesion cleavage, specifically RAD21 cleavage, is mediated by Separase (*ESPL1*), a protease that is normally held in check by Securin (*PTTG1*), which is rapidly degraded via a ubiquitin-mediated process orchestrated by APC/C-CDC20.

DNA re-sequencing efforts have determined that defects in cohesin and cohesion-related genes are implicated in various syndromes and diseases including cancer. Defects in *NIPBL*, *SMC1A*, and *SMC3* for example, are pathogenic in Cornelia de Lange syndrome, a rare genetic disorder associated with growth and cognitive impairment and a shortened lifespan (reviewed in [19]). Interestingly, individuals with Cornelia de Lange syndrome do not appear to have a predisposition to develop cancer, but this may be due to the shortened lifespan associated with a severe disease state. Still, rare cases of Wilm’s tumors and liver hemangioendothelioma have been identified in autopsies [20]. Somatic alterations in cohesion-related genes (e.g. *SMC1A*, *SMC3*, *STAG2*, *RAD21*, etc.) have however, been identified in a large number of CIN tumor types. Homozygous deletions, amplifications and single nucleotide polymorphisms, which presumably impact expression and/or function and ultimately sister chromatid cohesion have been identified in various cancers including breast, ovarian, colorectal, lung cancer, melanoma, Ewing’s sarcoma, acute myeloid leukemia, myeloid diseases and endometrial cancers [2,7-9,21-28]. Indeed, we recently demonstrated that diminished expression of a subset of these genes caused aberrant mitotic cohesion and CIN [7]. Collectively, these data suggest that altered expression and/or function of key cohesion-related genes are pathogenic events that underlie CIN and thus contribute to the development of cancer. However, it is currently unclear how pervasive and prevalent aberrant cohesion is within tumors, specifically within those that frequently exhibit CIN such as Hodgkin lymphoma (HL).

HL is a malignant proliferation of lymphoid cells that frequently exhibits CIN and extensive cytogenetic analyses have routinely identified numerical (and structural) changes in chromosomes within both HL patient samples and cell lines [29-31]. Although there is evidence implicating aberrant telomere biology in the etiology of HL [32-35], the prevalence of aberrant sister chromatid cohesion in HL has never been examined. In the present study, we examined the prevalence of aberrant sister chromatid cohesion within five common HL cell lines and a lymphocyte control. Using cytogenetic techniques, we first established the chromosome distribution profile and modal chromosome number for each line, and subsequently interrogated mitotic spreads for the presence of aberrant cohesion. Surprisingly, all HL lines exhibited aberrant cohesion and strikingly two lines, L-540 and HDLM-2, harbored cohesion defects in ~50% of all mitotic spreads examined. To identify the underlying mechanism that accounts for the aberrant cohesion, the localization and expression pattern of six key cohesion proteins, SMC1A, SMC3, STAG2, RAD21, Securin and Separase, was evaluated. Although all proteins exhibited the expected nuclear localization patterns within each line examined, there was significant variability in the level of RAD21 expression. Of particular interest, was the observation that RAD21 exhibit the greatest variation within these lines and in fact, it was lowest within L-540 and highest within HDLM-2 cell lines. Our results demonstrate that aberrant cohesion is a common feature of all five HL lines evaluated, and further suggest that aberrant RAD21 expression is a causative agent that contributes to the CIN phenotype, particularly within the L-540 and HDLM-2 cell lines. Collectively, our data support recent findings implicating aberrant cohesion as a significant pathogenic factor in the development of various tumor types, which now includes HL.

## Methods

### Cell lines and culture

The five HL cell lines, L-1236 (DSMZ; ACC-530), KM-H2 (DSMZ; ACC-8), L-428 (DSMZ; ACC-197), L-540 (DSMZ; ACC-72), and HDLM-2 (DSMZ; ACC-17) were cultured in RPMI-1640 (HyClone) medium supplemented with 10% fetal bovine serum (Sigma) in humidified incubator maintained at 37°C with 5% CO_2_. Cell lines were authenticated on the basis of recovery, viability, growth, and morphology. A blood sample was obtained with consent from a healthy volunteer by venipuncture and drawn into collection tubes containing heparin (BD Biosciences). Written informed consent was obtained, as approved by CancerCare Manitoba (Manitoba Institute of Cell Biology) and the University of Manitoba Ethics Review Committees in accordance with the Declaration of Helsinki. Control lymphocytes were isolated from the blood sample. The sample was centrifuged for 10 minutes at 1,500 rpm, and lymphocytes were collected and added to 30 ml of culture medium (RPMI-1640) supplemented with 10% fetal bovine serum containing penicillin/streptomycin. To stimulate proliferation of lymphocytes so that mitotic preparations could be generated, phytohemagglutinin (5 μg/ml; Invitrogen) was added to lymphocytes for 72 h (37°C).

### Mitotic chromosome spreads and enumeration

To enrich for mitotic chromosomes, asynchronous and subconfluent cells were treated with KaryoMax colcemid (0.1 mg/ml; Invitrogen) for 2 h prior to harvesting. Mitotic chromosome spreads were prepared as described previously [7], with the hypotonic (75 mM KCl) treatment extended to 15 minutes. Slides containing chromosome spreads were counterstained in Vectashield (Vector Laboratories) containing DAPI and a minimum of 300 mitotic spreads of either control or each HL cell line were imaged. Imaging was performed on an AxioImager 2 (Zeiss) equipped with an AxioCam HR charge-coupled device (CCD) camera (Zeiss) and a 63× oil immersion plan-apochromat lens (1.4 numerical aperture). Images were acquired with AxioVision software and saved as 16-bit Tif images. Images were enumerated and the total number of chromosomes from each mitotic spread was manually determined. All data were exported into Prism v6 (GraphPad) where the modal chromosomal number and chromosome distribution frequencies were determined. The normal chromosomal distribution range for each cell line was operationally defined as the chromosomal distribution observed between the 25^th^ and 75^th^ percentiles of the total distribution.

### Cohesion assay

Mitotic chromosome spreads were generated and imaged as detailed above and cohesion was evaluated at the primary constriction (i.e. centromere) as previously described [7]. Similar assays have been used previously to investigate sister chromatid cohesion. Cohesion was categorized into normal (cohesed) or aberrant (primary constriction gap; PCG). PCG is defined as a visually appreciable gap between the sister chromatids within the DAPI channel. Chromosome spreads exhibiting PCGs were further classified into one of three categories based on the severity and frequency of the PCGs within a given mitotic spread. We define the 3 aberrant PCG categories as; 1) PCG_I_ (mild), 1–4 chromosomes exhibit PCGs but are still in close proximity, 2) PCG_II_ (moderate), ≥5 chromosomes exhibit PCG but are still in close proximity, and 3) PCG_III_ (severe), all cohesion appears to be lost as chromatids appear randomly distributed throughout the spread). Finally, the frequencies of normal and aberrant cohesion were calculated and tables and graphs were generated.

### Indirect immunofluorescence

Indirect immunofluorescence labeling of cells was performed as detailed previously [36,37] with slight modifications. Briefly, asynchronous cells were harvested by centrifugation (180×g for 5 minutes), counted with a Coulter counter (BD), and resuspended in PBS to a final concentration of 10^7^ cell/ml. Approximately 10^6^ cells were spun (250 rpm; 5 minutes) onto glass slides using a Shandon CytoSpin 4 (Thermo Scientific). Cells were fixed with 4% paraformaldehyde, permeablized, immunofluorescently labeled and mounted in Vectashield (Vector Labs) containing DAPI. A list of the primary antibodies and working dilutions are indicated in Additional file 1: Table S1. All primary antibodies were visualized through the addition of either goat anti-rabbit Alexafluor488 (Invitrogen), goat anti-mouse Cy3 (Jackson ImmunoResearch Laboratories, Inc.), or donkey anti-goat FITC (Jackson ImmunoResearch Laboratories, Inc.) antibodies diluted at 1:200. Images were acquired as above and representative images were generated in Imaris and exported into Photoshop CS6 (Adobe) where panels were assembled.

### Immunoblot analysis

Immunoblots were performed on proteins extracted from lymphocytes obtained from healthy donors, and asynchronous and subconfluent cells of all five HL cell lines as described previously [38]. A list of the primary antibodies and working dilutions are indicated in Additional file 1: Table S1. Anti-rabbit (1:15,000), anti-mouse (1:10,000) or anti-goat (1:10,000) antibodies conjugated with horseradish peroxidase (Jackson ImmunoResearch Laboratories, Inc.) were employed to detect the primary antibodies by standard chemiluminescence. Blots were stripped and reblotted with an anti-α-tubulin mouse monoclonal antibody (Abcam ab7291 at 1:10,000) as a loading control. Figures were assembled in Photoshop and ImageJ (gel analyzer tool) was employed to determine the relative signal intensities of bands in a semi-quantitative fashion. All semi-quantitative data were normalized to the corresponding α-tubulin loading control for each lane and the relative fold differences in expression were determined.

## Results

### HL cell lines are aneuploid and exhibit a CIN phenotype

As CIN is a dynamic process that drives karyotypic evolution, it was first essential to characterize the chromosome number and distribution for each cell line and control employed. We purposely limited our study to five commonly employed HL cell lines L-1236, KM-H2, L-428, L-540 and HDLM-2, as each is reported to have an aneuploid (i.e. CIN) karyotype [39-41], while lymphocytes isolated from a normal, healthy donor were used as the control and are expected to have normal cohesion and a diploid karyotype. Mitotic chromosome spreads were generated as detailed in Methods, and chromosomes were enumerated from a minimum of 300 spreads (Table 1). As expected, all five HL cell lines were aneuploid relative to the diploid control, with the modal chromosome numbers ranging from hypodiploid for HDLM-2 (modal number = 36 chromosomes), to hypotriploid for L-540 (56 chromosomes), KM-H2 (61 chromosomes), and L-1236 (65 chromosomes), to hypotetraploid for L-428 (91 chromosomes). Moreover, the total chromosome distribution ranges were dramatically larger for each of the five HL cell lines relative to the control as were the normal distribution ranges (as defined by the 25^th^ to 75^th^ percentile), thus confirming the five HL cell lines exhibit CIN phenotypes and are aneuploid.

**Table 1:** Characterization of chromosome distribution frequencies in various HL cell lines

**Cell line**	**N**^**A**^	**Total chromosome range**	**Modal chromosome number**	**Normal chromosome range**^**B**^
Control	300	46-47	46	46
HDLM-2	300	31-123	36	36-54
KM-H2	325	41-143	61	58-65
L-428	319	45-186	91	86-94
L-540	348	41-148	56	56-74
L-1236	310	41-138	65	59-66

^A^Total number of mitotic chromosome (Chr.) spreads evaluated.

^B^Normal Chromosome Range is defined as the 25^th^ to 75^th^ percentile of the total chromosome distribution range on the given cell line.

### Aberrant sister chromatid cohesion is common to all HL cell lines examined

**Figure 1:**
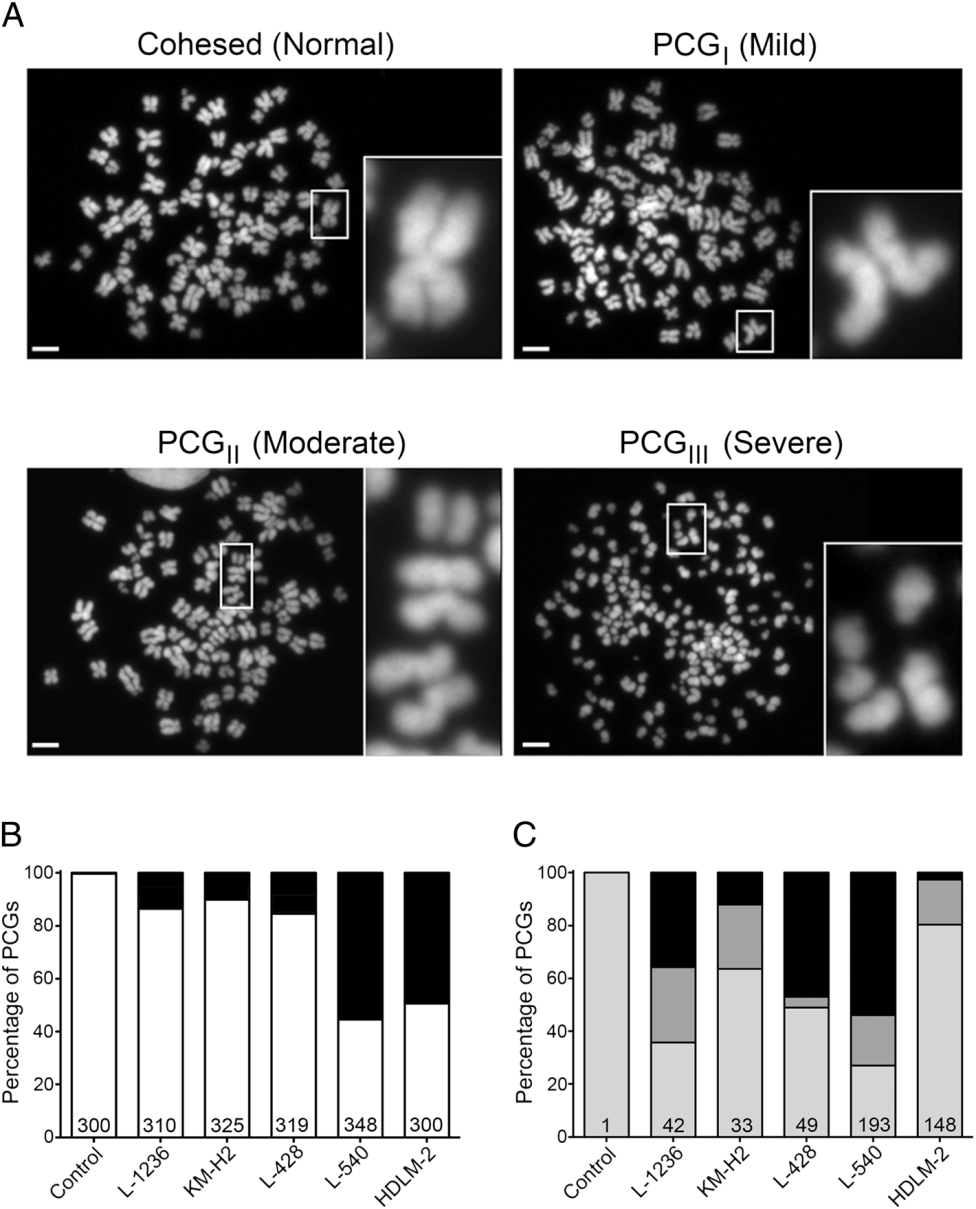
**HL cell lines exhibit cohesion defects. (A)** Representative images of mitotic chromosome spreads from the L-540 cells demonstrating normal primary constriction cohesion (Upper left) and the various categories of aberrant cohesion; PCG_I_ (Mild), PCG_II_ (Moderate), and PCG_III_ (Severe). Each cohesion category (quadrant) contains a low-resolution DAPI image of the entire mitotic spread. Each spread contains a white bounding box that defines the region magnified and presented on the right-hand side of each quadrant. Scale bar represents 10 μm. **(B)** Graphs depicting the total fraction of cells with aberrant cohesion; white (normal), black (aberrant). The total number of mitotic spreads evaluated for each cell line is indicated at the bottom of each column. **(C)** Graphical depiction for *only* the fraction of mitotic spreads with cohesion defects. The frequencies of defects are classified according to three different aberrant cohesion categories (e.g. PCG_I_ - light gray; PCG_II_ - dark gray; and PCG_III_ - black). The total number of mitotic spreads exhibiting aberrant cohesion is indicated at the base of each column.

Having established that each of the HL cell lines exhibit CIN, we now wished to examine whether sister chromatid cohesion, as measured by primary constriction cohesion, was normal or aberrant within each line. We have previously defined aberrant cohesion or primary constriction gaps (PCG) as a clear and distinct separation within the DAPI signal occurring between the sister chromatids at the centromere, the site of the primary constriction (Figure 1A; top left quadrant) [7]. PCGs were manually assessed in a minimum of 300 mitotic spreads generated from each HL cell line and control. Surprisingly, all HL cell lines had striking increases in PCG frequencies relative to the lymphocyte control (Figure 1B and Table 2) that range from 10.2% in KM-H2 to 55.4% in L-540. Of particular note, ~50% of all spreads evaluated from L-540 (55.4%) and HDLM-2 (49.3%) exhibited PCGs, which is ~160-fold greater than the control in which only a single chromosome within a single spread exhibited a mild PCG phenotype.

**Table 2:** Frequency of primary constriction gaps in various HL cell lines

**Cell line**	**N**^**A**^	**Normal cohesion (%)**	**Primary constriction gap (PCG) (%)**			
			**PCG**_**I**_	**PCG**_**II**_	**PCG**_**III**_	**Total**^**B**^
Control	300	99.7	0.3	0.0	0.0	0.3
HDLM-2	300	50.7	39.7	8.3	1.3	49.3
KM-H2	325	89.8	6.5	2.5	1.2	10.2
L-428	319	84.7	7.5	0.6	7.2	15.3
L-540	348	44.6	14.9	10.6	29.9	55.4
L-1236	310	86.5	4.8	3.9	4.8	13.5

^A^N; total number of chromosome spreads evaluated.

^B^Refers to the total number of cohesion defects (i.e. PCG_I_ + PCG_II_ + PCG_III_).

To further characterize the severity of the PCG phenotypes, all spreads exhibiting aberrant cohesion were further classified into three distinct sub-categories (Figure 1A) ranging from mild (PCG_I_) to moderate (PCG_II_) to severe (PCG_III_) (see Methods for definitions). Interestingly, all five HL cell lines harbored mitotic spreads within each of the three aberrant cohesion categories (Table 2), while only one chromosome from a single spread exhibited a mild cohesion defect (i.e. PCG_I_) representing a baseline frequency of ~7.2x10^-5^ cohesion defects/chromosome (1/[300 spreads × 46 chromosomes]). Further scrutiny of only those mitotic spreads with PCGs revealed that with the exception of L-540, PCG_I_ was the most prevalent aberrant category observed and ranged from 35.5% in L-1236 to 80.5% in HDLM-2 (Figure 1C). In L-540, the most prevalent aberrant category was PCG_III_ (53.9%). Of particular note is the inverse relationship that was observed between the two cell lines with the greatest abundance of aberrant cohesion (L-540 and HDLM-2). The most frequently observed aberrant category in L-540 is PCG_III_ (severe) while in HDLM-2 it is PCG_I_ (mild), suggesting that the aberrant mechanism(s) underlying the cohesion defects may be different.

### Altered RAD21 expression is associated with elevated cohesion defects

**Figure 2:**
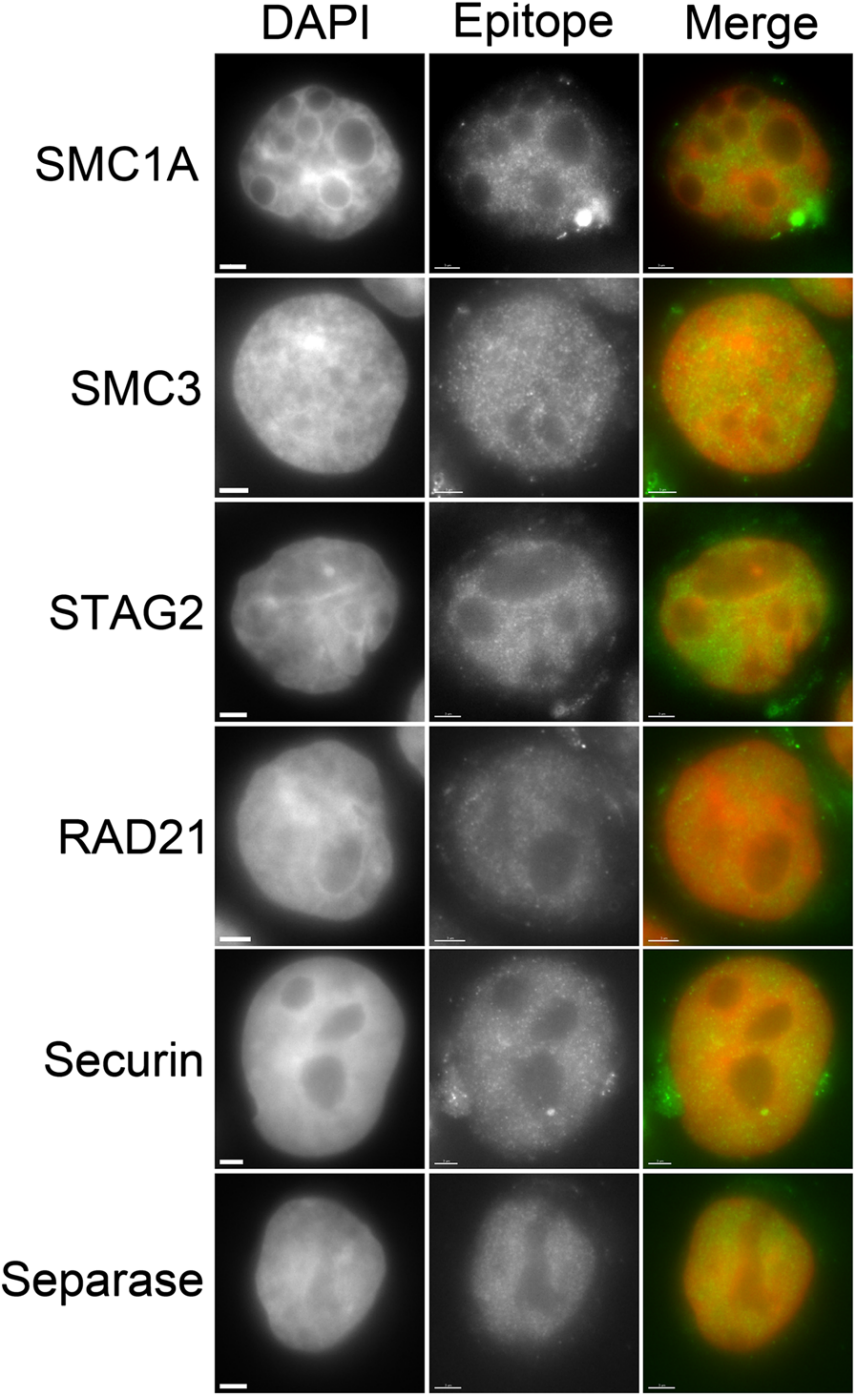
**Spatial localization of cohesion-related proteins in L-540 cells.** Indirect immunofluorescence was employed to determine the spatial localization of six proteins involved in sister chromatid cohesion (SMC1A, SMC3, STAG2, RAD21, Securin and Separase). Presented are representative high-resolution (63×) images obtained from L-540 cells in interphase. Nuclei were counterstained with DAPI and immunofluorescently labeled for the epitope indicated on the left. Scale bar represents 3 μm.

We now wished to investigate the underlying mechanisms that account for the aberrant cohesion observed within the HL lines, with a particular focus on HDLM-2 and L-540 due to the high prevalence of PCGs (~50%) within those lines. We purposefully restricted our analysis to six candidates that are central to cohesion, and that have been implicated in the pathogenesis of other tumor types [7,42,43]. Included in this list are the four members of the cohesin complex, SMC1A, SMC3, RAD21 and STAG2, as well as Securin and Separase, which regulate cohesin cleavage. As an initial step, we first surveyed the Broad-Novartis Cancer Cell Line Encyclopedia (http://www.broadinstitute.org/ccle/home) and failed to identify a single somatic mutation within any of the six candidate genes in any of the five HL cell lines (data not shown). However, the possibility remained that the HL lines we employed may have accrued somatic and/or epigenetic alterations that could impact protein localization, expression and/or function. Accordingly, we performed indirect immunofluorescence to determine the expression and localization pattern of each candidate within the five HL lines. Previous studies from various tumor types have shown that each candidate normally exhibits a nuclear-enriched staining pattern within interphase nuclei. Here we confirm a similar, nuclear-enriched, focal staining pattern within each of the five HL cell lines. For example, in L-540 (Figure 2) and HDLM-2 (Additional file 1: Figure S1) each candidate was enriched within interphase nucleus, but noticeably absent from nucleoli.

**Figure 3:**
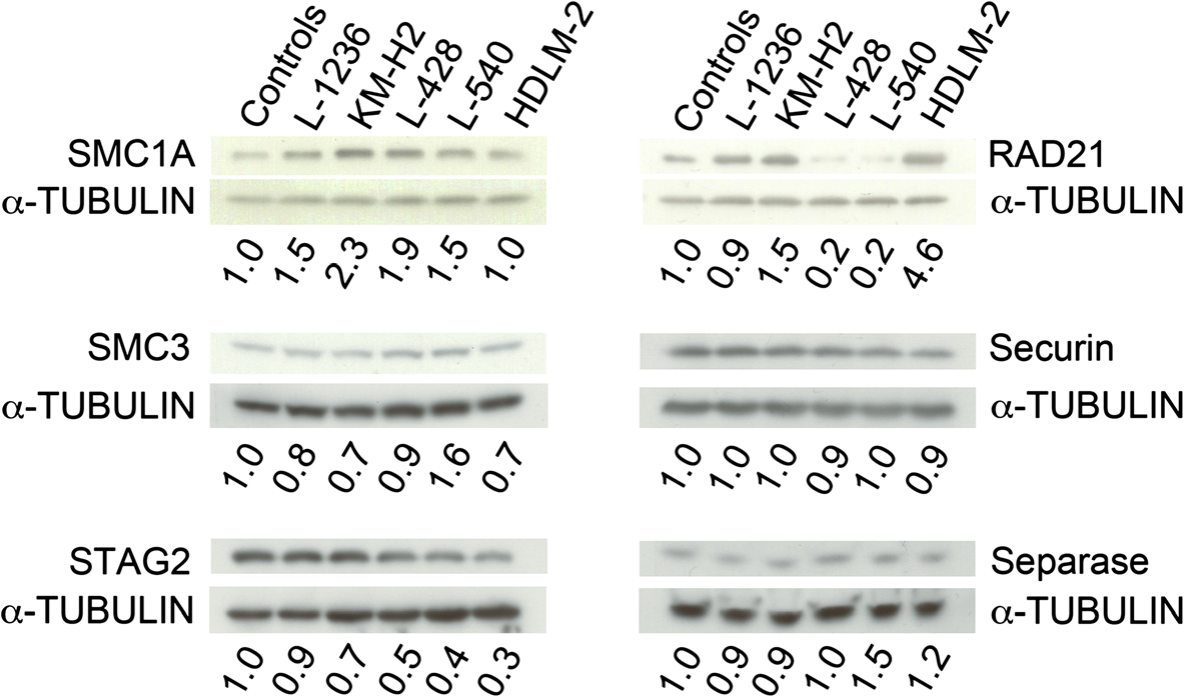
**Expression levels of cohesion-related proteins in HL cell lines.** Representative immunoblots depicting the relative expression levels of 6 specific cohesion-related proteins in a lymphocyte control and five HL cell lines (indicated at top) with α-tubulin serving as a loading control. Semi-quantitative values for each cohesion epitope were determined and adjusted for unequal loading by generating a ratio with corresponding loading control. To further facilitate comparisons between cell lines, all semi-quantitative values were normalized to the lymphocyte control (1^st^ lane in each gel; values set to 1.0) with the relative expression levels indicated.

The above observations indicate that protein mis-localization and epigenetic silencing (i.e. complete silencing) of the candidates are unlikely causes of the cohesion defects observed within these HL lines. However, they do not address the possibility that hypo- or hyper-morphic expression may occur and be a pathogenic event, as has previously been reported in other tumor types [7,21-24]. Consequently, we evaluated the expression levels of all six candidates within each of the HL cell lines using semi-quantitative Western blots (Figure 3). For comparison purposes, the abundance of each protein was first quantified, normalized to the corresponding loading control, and is presented relative to the lymphocyte control, which was set to 1.0. Interestingly, five of the candidates, SMC1A, SMC3, STAG2, Securin and Separase, were expressed in similar amounts in all five lines investigated. In fact, the overall range of protein expression varied from a low of 1.1-fold for Securin to 3.3-fold for STAG2. However, the expression of RAD21 was considerably greater amongst the five HL lines and 23-fold different – it was lowest in L-428 and L-540 at 0.2-fold and highest in HDLM-2 at 4.6-fold when compared to the lymphocyte control. Of particular note is the fact that the two lines with the highest prevalence of aberrant cohesion (~50%), were at the minimal (L-540) and maximal (HDLM-2) extremes of RAD21 expression. It is also of interest to note that STAG2 expression is diminished within the HDLM-2 (0.3-fold) and L-540 (0.4-fold) cells relative to the lymphocyte control.

## Discussion

Evidence has accrued that implicates aberrant sister chromatid cohesion as a pathogenic event that causes CIN, and contributes to the development and progression of cancer [7]. Despite this information, the prevalence of aberrant cohesion within HL, a tumor type that frequently exhibits CIN, has never been determined. Because of this, we purposefully limited the current study to investigations of aberrant cohesion within HL, and thus, we did not attempt to address the role(s) aberrant cohesion may have on gene expression and/or DNA repair. In the current study, we evaluated a panel of five commonly employed aneuploid HL cell lines and show that each exhibits cohesion defects ranging from 10.2% to 55.4%. Strikingly, two lines, L-540 and HDLM-2, harbor defects in ~50% of all mitotic spreads evaluated. Subsequent semi-quantitative Western blot analyses determined that RAD21 expression varied greatly amongst all HL lines but were highest in HDLM-2 and lowest in L-540.

Collectively, our data demonstrate that cohesion defects are a common feature of all HL lines investigated, but are particularly prevalent within the HDLM-2 and L-540. Our study also suggests that aberrant RAD21 expression may contribute to the high levels of aberrant cohesion in HDLM-2 and L-540 cells. Although our analyses cannot preclude *de novo* mutations that impact the functions encoded by the six candidate genes evaluated, data gleaned from the Broad-Novartis Cancer Cell Line Encyclopedia [44] failed to identify a single somatic mutation in any of the six genes within any of the HL cell lines. Moreover, our expression and localization data show that each protein is expressed and exhibits the expected nuclear-enriched localization pattern indicating that epigenetic silencing and/or mis-localization of the six candidates cannot account for the cohesion defects we observe. It should be noted however, that many additional proteins impact sister chromatid cohesion including cohesion loaders, kinases and those involved in cohesion establishment. For example, we previously identified novel roles for CDC4/FBXW7 (a classical cell cycle regulator) and MRE11A (a DNA repair protein) in cohesion [7]. Thus, it remains formally possible that aberrant expression of additional cohesion-related proteins may contribute to the aberrant cohesion and CIN observed in the HL cell lines. Indeed, the differences in the prevalence and severity of the PCGs between these lines (see Table 2; predominantly PCG_I_ in HDLM-2 vs. predominantly PCG_III_ in L-540) suggest that the underlying aberrant mechanism(s) accounting for the cohesion defects are likely to be distinct. Nevertheless, our findings support a growing body of evidence implicating aberrant cohesion as a contributing and driving factor in the development of various cancers, which based on the current findings, can be expanded to include HL.

It is now becoming apparent that altered expression, function, and/or localization of cohesin and cohesion-related proteins causes aberrant cohesion, CIN and is correlated with various disease states. For example, somatic alterations in genes that regulate sister chromatid cohesion have been identified in a number of tumor types including breast, colorectal lung and ovarian cancers, Ewing’s sarcoma, melanoma, acute myeloid leukemia, and myeloid diseases suggesting that aberrant expression contributes to tumorigenesis [7,21-24,26-28,42,44-46]. Four large-scale, gene re-sequencing efforts by the Cancer Genome Atlas (TCGA) Network identified non-synonymous single nucleotide polymorphisms (nsSNPs), homozygous deletions and gene amplifications in breast [21], colorectal [22], lung [24] and ovarian [23] cancers (Table 3). Most relevant to the current study is the finding that *RAD21* is frequently amplified in two of the four tumor types evaluated. *RAD21* is amplified in 13.9% (67/482 tumors) of breast tumors evaluated [21], while it is amplified in 18.0% (57/316 tumors) [23] of ovarian tumors. In breast cancer, correlations have been identified between enhanced RAD21 expression [47] (and the presence of specific nsSNPs [48]) and overall breast cancer risks, and RAD21 over-expression is associated with poor prognosis and the acquisition of drug resistance [47]. Aberrant RAD21 mRNA expression has also been examined in endometrial cancers where Supernat et al. [49] noted elevated expression strongly correlated with more advanced tumor stages and grades. Finally, *RAD21* is amplified and over-expressed in prostate cancer [50], and its expression is correlated with invasion and metastasis in oral squamous cell carcinomas [51]. In agreement with these findings, our data show that RAD21 expression levels are elevated in HDLM-2 and are associated with a high prevalence of aberrant cohesion and CIN. Our results also agree with recent mRNA expression data indicating that RAD21 levels are frequently increased in liquid tumors (Additional file 1: Figure S2). For example, publically available data from the CCLE_expression_Entrez_2012-10-18.res: Gene-centric RMA-normalized mRNA expression data (http://www.broadinstitute.org/ccle) indicate that RAD21 mRNAs levels from the HL cell lines rank 5^th^ amongst the 37 different tumor types evaluated (Additional file 1: Figure S2). Collectively, these data strongly suggest that RAD21 over-expression may be a pathogenic event in a variety of tumor types, including HL.

**Table 3:** **Somatic alteration in cohesion-related genes in cancer**^**A**^

**Gene**	**Percentage of somatic alterations**^**B **^**in**											
	**Breast (482**^**C**^**)**			**Colorectal (212**^**C**^**)**			**Lung (178**^**C**^**)**			**Ovarian (316**^**C**^**)**		
	**nsSNP**	**Del**	**Amp**	**nsSNP**	**Del**	**Amp**	**nsSNP**	**Del**	**Amp**	**nsSNP**	**Del**	**Amp**
*SMC1A*	0.8	0.0	0.0	4.2	0.0	0.0	1.1	0.0	0.0	1.3	0.0	0.0
*SMC3*	0.2	0.2	0.0	1.4	0.9	0.0	2.8	0.6	0.0	0.3	0.0	0.0
*RAD21*	0.6	0.0	13.9	2.8	0.0	1.9	1.7	0.0	0.0	0.3	0.6	18.0
*STAG2*	1.2	0.0	0.0	2.8	0.0	0.0	3.4	0.0	0.0	0.9	0.0	0.0
*PTTG1*^B^	0.0	0.4	0.2	0.5	0.0	0.0	1.1	0.6	0.0	0.0	0.0	0.9
*ESPL1*^C^	1.5	0.0	0.2	3.3	0.0	0.0	4.5	0.0	0.6	0.0	0.3	0.6

^A^Only the Cancer Genome Atlas (TCGA) data is shown [21-24].

^B^non-synonymous single nucleotide polymorphism (nsSNP), homozygous deletion (Del), gene amplification (Amp).

^C^The total number of tumors sequenced is indicated within parentheses.

It is now becoming clear that RAD21 is an excellent and attractive molecule to study for several reasons. From a tumorigenic perspective altered RAD21 expression is associated with a number of tumor types – over-expression is associated with advanced tumor stage and grade, enhanced invasion and metastasis, and the acquisition of multidrug resistance. However, from a therapeutic targeting perspective, RAD21 is also an attractive candidate. For example, Atienza et al. [52] recently showed that RAD21 suppression decreases cell growth and enhances cytotoxicity to two chemotherapeutic agents, suggesting that chemically targeting RAD21 may hold therapeutic potential. Consequently, tumors that over-express RAD21, such as HDLM-2, may be sensitive to RAD21 suppression. Thus, of the four members of the cohesin complex members, RAD21 is emerging as a lead candidate that may predict tumor behavior and therapeutic resistance, but it may also represent a candidate therapeutic target. In light of these findings we believe that aberrant RAD21 expression, particularly over-expression in HDLM-2 and perhaps underexpression in L-540 (and L-428), has a role in the development and progression of various tumor types, which we have expanded to include HL. Future studies will be required to elucidate the specific mechanism(s) by which RAD21 and other cohesion genes contribute to the development of various tumor types such as HL.

## Conclusions

Despite evidence suggesting that aberrant sister chromatid cohesion is a driver of the tumorigenic process, its prevalence within HL is unknown. Our data demonstrate that aberrant cohesion is common to all five HL cell lines evaluated. They further show that aberrant cohesion is present in ~50% of all spreads examined for L-540 and HDLM-2, which also exhibit aberrant RAD21 expression. Accordingly, we conclude that aberrant cohesion is a common feature within HL cell lines. Based on this study and supporting data from other tumor types, we further conclude that aberrant RAD21 expression is a strong candidate that contributes to the development of aberrant cohesion, CIN and tumor development in these cell lines and perhaps HL. Thus, it will be important to extend the current findings of this study into patient samples to characterize the prevalence of aberrant cohesion and aberrant RAD21 expression, and establish their putative roles as causative agents in the development of HL.

## Abbreviations

(CIN): Chromosome instability; (HL): Hodgkin lymphoma; (nsSNP): Non-synonymous single nucleotide polymorphism; (PCG): Primary constriction gap; (DSMZ): German Collection of Microorganisms and Cell Cultures GmbH (Deutsche Sammlung von Mikroorganismen und Zellkulturen GmbH); (CO2): Carbon dioxide; (KCl): Potassium chloride; (DAPI): 4',6-diamidino-2-phenylindole; (PBS): Phosphate buffered saline; (CCD): Charge-coupled device.

## Competing interests

The authors declare that they have no competing interests.

## Authors’ contributions

BVS/KJM conceived the study. BVS/ZL carried out the cytogenetic work. BVS/KJM analyzed and interpreted the results. BVS/KJM wrote the manuscript. All authors read and approved the final manuscript.

## Pre-publication history

The pre-publication history for this paper can be accessed here:

http://www.biomedcentral.com/1471-2407/13/391/prepub

## Supplementary Material

Additional file 1: Figure S1 Spatial Localization of Cohesion-Related Proteins in HDLM-2. Figure S2: Gene Expression Data for RAD21 in Human Cancer Cell Lines. Table S1: Antibodies Dilutions Employed in this Study. Click here for file
