# Supplementary material for: Sister chromatid cohesion defects are associated with chromosome instability in Hodgkin lymphoma cells
Source: BMC Cancer. 2013 Aug 20;13:391. doi: 10.1186/1471-2407-13-391 (PMC3751861; doi:10.1186/1471-2407-13-391)
Supplement: Additional file 1: Figure S1 — Spatial Localization of Cohesion-Related Proteins in HDLM-2. Figure S2: Gene Expression Data for RAD21 in Human Cancer Cell Lines. Table S1: Antibodies Dilutions Employed in this Study. [file 1471-2407-13-391-S1.pdf]

ADDITIONAL FILES

Supplementary Figure 1: Spatial Localization of Cohesion-Related Proteins in HDLM-2.

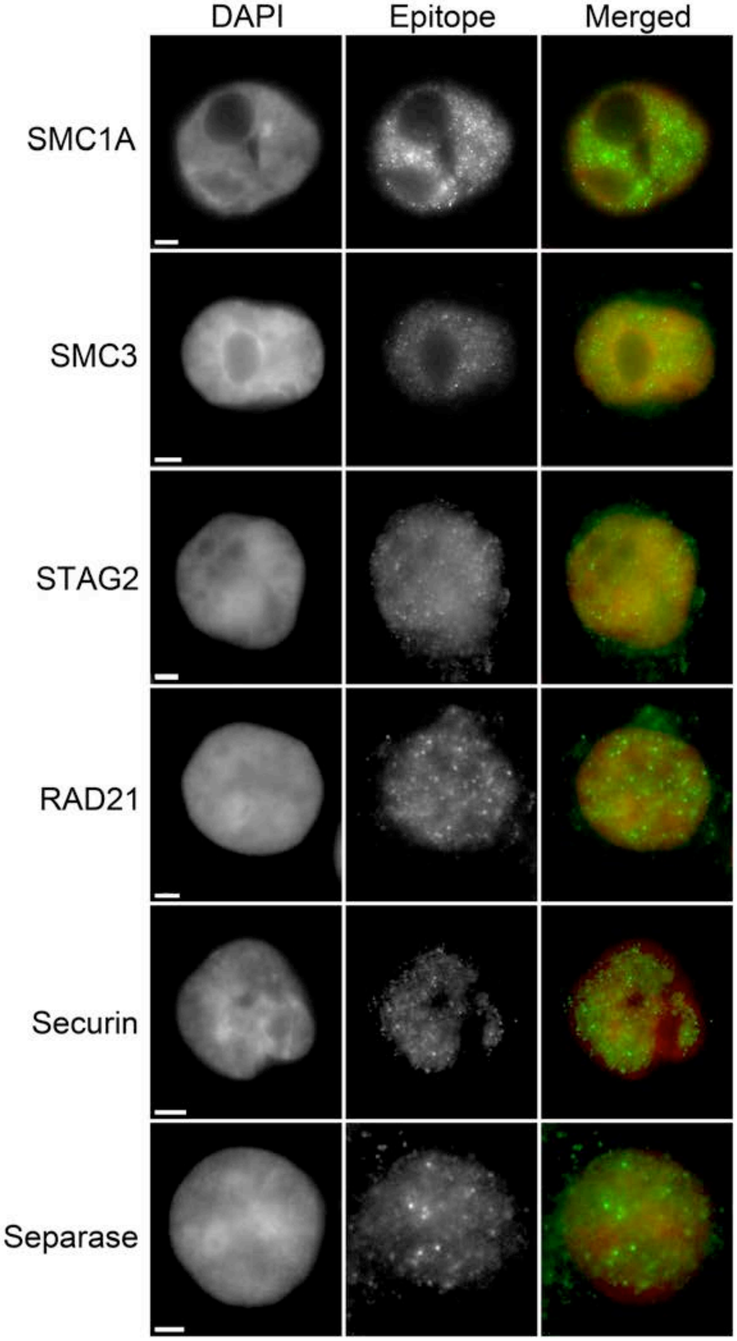

Supplementary Figure 1: Spatial Localization of Cohesion-related Proteins in HDLM-2. Indirect immunofluorescence was employed to evaluate the localization of six proteins central to sister chromatid cohesion in HDLM-2. Presented are representative low-resolution images obtained from HDLM-2 cells. Nuclei were counterstained with DAPI and immunofluorescently labeled for the epitope indicated. Scale bar represents 20μm.

Supplementary Figure 2: Gene Expression Data for RAD21 in Human Cancer Cell Lines.

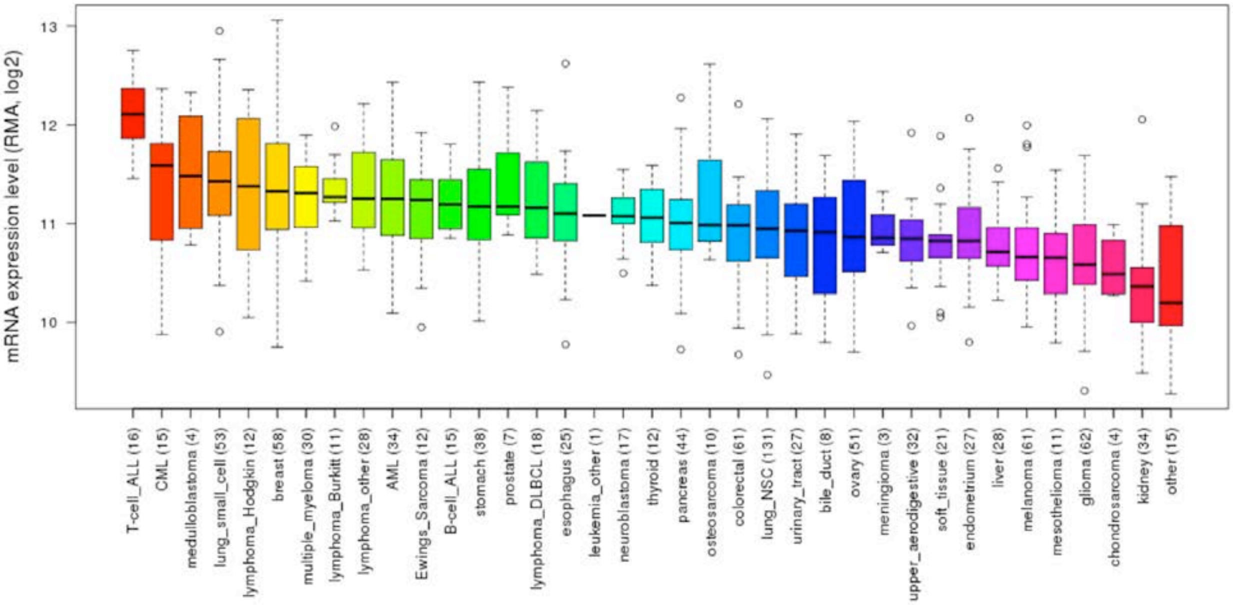

Supplementary Figure 2: Gene Expression Data for RAD21 in Human Cancer Cell Lines.
Graphical depiction of the publically available mRNA expression data for *RAD21* in various cancer cell lines (*x*-axis): numbers in brackets indicate number of cell lines evaluated within each tumor type. All data is extracted from the CCLE_Expression_Entrez_2012-10-18.res :Gene-centric RMA-normalized mRNA expression data (http://www.broadinstitute.org/ccle/home).

Supplementary Table 1: Antibodies Dilutions Employed in this Study.

**Epitope**	**Source**	**Cat. No.**	**IIF Dilution[A]**	**WB Dilution[B]**
SMC1A	Abcam	ab9262	1:2,000	1:7,500
SMC3	Abcam	ab9263	1:500	1:3,000
RAD21	Abcam	ab16473	1:200	1:2,000
STAG2	Abcam	ab4463	1:200	1:1,000
Securin	Abcam	ab26273	1:200	1:1,000
Separase	Abcam	ab3762	1:200	1:1,000

[A]Indirect Immunofluorescence (IIF)
[B]Western Blot (WB)
